# The effect of a medication safety reminder letter for high-risk patients under a universal health insurance scheme- A pilot study

**DOI:** 10.1016/j.puhip.2025.100684

**Published:** 2025-12-11

**Authors:** Shou-Hsia Cheng, Yafang Tsai

**Affiliations:** a Institute of Health Policy and Management, College of Public Health, National Taiwan University, Taipei, Taiwan, ROC; b Department of Health Policy and Management, Chung Shan Medical University, Taichung, Taiwan, ROC; c Department of Medical Management, Chung Shan Medical University Hospital, Taichung, Taiwan, ROC; d Institute of Health Policy and Management, and Population Health Research Center, Taiwan, ROC

**Keywords:** Medication duplication, Nudging, Patient activation, Information, Taiwan

## Abstract

**Objectives:**

Nudge interventions have been applied to change patients' health behavior in the areas of smoking cessation and healthy food choices. Patient activation is one of the key elements in the self-management of health. This study explored whether a medication safety reminder for high-risk patients can prompt doctors to reconcile patients’ prescriptions and examine the role of patient activation.

**Study design:**

This was a cross-sectional study.

**Methods:**

This study selected eleven thousand subjects from the list of patients with duplicated medication in 2019 provided by the Taiwanese single-payer insurance scheme. Postal reminder letters were sent to the patients. After a month, questionnaires were sent out to ask patients whether they had consulted their doctors after receiving the medication reminder letter, and the doctors checked or revised their prescriptions. A total of 841 completed questionnaires were received, and 34.8 % of them had asked their doctors to check the prescription.

**Results:**

The results from regression models revealed that patients with higher patient activation had a higher rate (odds ratio [OR] = 2.617) of asking their doctor to check the prescription (p < 0.001) compared with those with lower patient activation.

**Conclusions:**

The present study shows that nudging intervention by the health insurer to the patients can prompt individuals to request healthcare providers to check their prescriptions. This may reduce healthcare resource waste and increase care safety.

## 1 Introduction

In order to improve the health status of the general population, health authorities tend to introduce programs with financial incentives to change people's behavior in the health sector. “Quit to win” for smoking cessation is a well-known example [1,2]. However, policies that involve financial incentives usually require a certain amount of budget, which limits the application of such policies. Scholars have suggested that applying nudges to change people's health behavior may achieve the same goal with minimal expenditures [3,4]. Nudges are defined as ”any aspects of the choice architecture that alter people's behavior predictably without forbidding any options or significantly changing their economic incentives” [5]. Hummel and Maedche [6] suggested that, based on the concept of choice architecture, effective nudging can be designed to induce changes in people's health behavior. Shumaker et al. have proposed methods for designing nudges, and providing a message is commonly used [7].

Nudge interventions have been widely applied to change patients' health behavior in the areas of smoking cessation and healthy food choices [[8], [9], [10]]. Furthermore, studies have used nudge interventions to change doctors' behavior. For example, Meeker et al. [11] asked doctors to sign a commitment to use antibiotics more cautiously when dealing with acute respiratory tract infections. Doctors who signed the commitment were found to use antibiotics more cautiously than those who did not. Caris et al. [12] investigated whether behavioral nudges, displayed as posters, can increase the use of alcohol-based hand rub. Research results have confirmed that reminder posters increase the subject's frequency of using such rubs, thereby improving doctors' hand hygiene. Bourdeaux et al. [13] explored the influence of a default setting in electronic prescribing systems on doctors' decision-making for acute care patients. They found that such care costs were lower after doctors used the system.

Many interventions have focused on adopting financial incentives in healthcare settings to change patients' or doctors' behavior, such as pay-for-performance programs [14,15]. In addition to financial incentives, shared decision-making benefits doctor-patient communication and may improve patients’ health literacy and care outcomes [16]. The World Health Organization (WHO) [17] has proposed a strategy called “Medication Without Harm,” which promotes medication communication between patients and healthcare providers. Patient activation could also increase the quality of care and improve patient-physician communication [18] and health-related outcomes [19].

Patient activation emphasizes patients' willingness and ability to take independent actions to manage their health and care [20]. ‘‘Patient activation’’ is a term used to describe the extent to which individuals can manage their healthcare [21]. Scholars defined “patient activation” as a patient's ability to handle health and health care [22,23]. Hibbard et al. [24] conceptualized patient activation as encompassing a range of elements essential in self-management that extend beyond any health behavior. They developed the Patient Activation Measure (PAM), a 4-level model of a patient's health behavior to measure patient activation. The lowest level indicates that the patient may not believe his/her role in self-care management is essential; the second level indicates that the patient lacks confidence or knowledge to take an active role; the third level indicates that the patient is beginning to be active; and the at highest level, patients actively attempt (even with difficulty) to maintain health management behaviors over time [21,25]. Previous studies have revealed that patient activation is significantly associated with medication adherence and health outcomes [19,26,27].

### 1.1 Taiwanese health system

Taiwan has implemented a universal, single-payer health insurance system since 1995 that covers 99.6 % of the population, and more than 95 % of hospitals and clinics are contracted with the insurer [28]. Under the mainly fee-for-service payment scheme, patients in Taiwan have a very high level of freedom in choosing healthcare providers without referral, leading to possible doctor-shopping behavior [29]. Consequently, on average, people in Taiwan visit their doctors about 15 times per year, and the occurrence of medication duplication is higher than in other countries [30].

To address the frequent and fragmented physician visits and potentially duplicated prescriptions, the single-payer National Health Insurance (NHI) Administration in Taiwan developed a medication duplication index in 2011 to monitor healthcare providers’ prescribing behavior. In 2013, the NHI Administration established a centralized medication record-sharing system (i.e., NHI PharmaCloud), which contains prescription records of each physician visit for every beneficiary under the NHI scheme, and the information was accessible to every contracted provider [30]. Since then, all contracted healthcare providers have been requested to check the medication information via the NHI PharmaCloud to avoid duplicate or inappropriate prescriptions for patients.

In 2016, the NHI Administration began to generate and provide a list of patients with duplicated medication to each healthcare provider as a reminder for medication safety improvement and cost containment. Then, a penalty was imposed in 2017; when a patient received duplicated medication from a provider, the drug expenses would be deducted accordingly from the claims. However, after several years of implementing the NHI PharmaCloud system, the problem of medication duplication persists among patients with multiple chronic conditions [31,32].

It seems that the financial incentives (penalty) to providers only lead to a limited effect in reducing the occurrence of medication duplication. The Taiwanese NHI Administration carried out a “postal reminder letter” project to improve patient medication safety as a nudging intervention tool for patients with duplicated medication. The reminder letter intervention project was conducted jointly with the research team at National Taiwan University, and the project has been approved by the Institutional Review Board at National Taiwan University Hospital (201905067RINB). The present study aimed to examine whether the reminder letters induce patients to request their doctors to check their prescription records; furthermore, this study also explored the influence of patient activation on patients’ actions to raise the request for checking prescription records.

## 2 Methods

### 2.1 The reminder letter intervention

The NHI administration has regularly generated a list of patients with duplicate prescriptions of 60 medications for each hospital and clinic since 2016. The reminder letter project used the list of patients between January and March 2019 as potential candidates for the intervention. We then randomly selected 11,000 patients with 14 days or more of duplicate medication for diabetes mellitus and hyperlipidemia for the study. The name tab for postal mailing was provided and processed by the NHI Administration; all of the subjects' identifiable information had not been revealed to the research team. A postal reminder letter was sent to the subjects during the first week of July 2019.

The reminder letter contains the following statements: The NHI Administration regularly analyzes the prescription records of patients with chronic conditions and notices that you (on the mailing list) have received duplicate medication in the first quarter of 2019. A duplicate prescription might be necessary for your condition, or you may have received the prescription for treating the same symptoms of different diseases from different healthcare providers. Taking duplicated medication may lead to sub-optimal health outcomes. We advise you to bring all your prescribed medicines with you next time you visit your doctor and ask the doctor to check the NHI PharmaCloud for all your medications.

### 2.2 The questionnaire survey

To understand the patients' reactions after receiving the reminder letters, the research team worked with the NHI Administration and conducted a questionnaire survey by postal mail to those reminder letter recipients one month later. From July 31 to August 2, 2019, the research team sent out 10,723 questionnaires (excluding 277 subjects whose reminder letters were returned to the NHI Administration due to invalid mailing addresses).

The questionnaire comprised three parts. The first part inquired about the patients' responses upon receiving the postal reminder letters. The items were: (1) Did you ask your doctor to check your prescription at the following physician visit after receiving the postal reminder letter? and (2) Did your doctor use the NHI PharmaCloud system to check your prescription? The second part of the survey collected the patients’ demographic characteristics, such as age, sex, education, marital status, and household income, as well as health status, such as self-perceived health, the number of physician visits in the past month, the number of hospitalizations in the previous year, and the number of medications taken every day.

Hibbard et al. [24] believe that patients' beliefs are an important factor affecting patients' enthusiasm for treatment and medication behavior. Considering the questionnaire length, our study selected three items to measure patients' medication beliefs to measure patient activation in the third part, according to a previous study by Hibbard et al. [24]. The three questions were (1) When all is said and done, I am the person who is responsible for managing my health condition; (2) I know what each of my prescribed medications does; and (3) I am confident that I can follow through on medical treatments I need to do at home. Patient activation was measured using a 4-point Likert scale with response anchors of *strongly disagree*, *disagree*, *agree*, and *strongly agree*.

### 2.3 Data analysis

Data analysis was conducted using SPSS Microsoft 22.0. The significance level was set α = 0.05. The categorical variables were presented as absolute (n) and relative (%) frequencies. A Chi-square analysis was used to investigate the effect of the reminder letter on the patient's reaction after receiving the letter. A multivariate linear regression was applied to analyze the impact of patient activation while controlling for other confounding factors.

## 3 Results

### 3.1 Demographic, socioeconomic characteristics, and health condition

A total of 841 completed questionnaires were received, with a response rate of 7.84 %. About 42.09 % of the participants were over 70 years old. About 65.6 % of the participants were male. The majority of participants earned an income between 10 and 49.99 thousand New Taiwan Dollars/per month (50.89 %). About 42.21 % of participants graduated from Junior high or Senior high school. About 26.28 % of the participants were single/others. 56.36 % of the participants reported good or very good health status. 75.27 % of the participants had been hospitalized in the previous year. About 80.14 % of the participants had visited a doctor in the past month. 42.58 % of the participants were taking five or more medication pills daily (Table 1).

**Table 1:** The characteristics of study subjects.

	The number of participants (%)
**Demographic factors**	
Age	
<59	222(26.40)
60-69	265(31.51)
>70	354(42.09)
Gender	
Male	552(65.6)
Female	289(34.4)
**Socioeconomic factors**	
Monthly income	
<NT$10000	70(8.32)
NT$10000-NT$49999	428(50.89)
>NT$50000	343(40.78)
Education	
Primary school	218(25.92)
Junior high/Senior high	355(42.21)
College	268(31.87)
Marriage status	
Married	620(73.72)
Single/Others	221(26.28)
**Health status**	
Reporting good or very good health	
Yes	474(56.36)
No	367(43.64)
Hospitalized in the previous year	
No	633(75.27)
Yes	208(24.73)
A physician visit in the past month	
No	167(19.86)
Yes	674(80.14)
Number of medications you take every day	
1–2	177(21.04)
3-4	306(36.38)
5 or more	358(42.58)

### 3.2 Simple association between patient characteristics and response to the reminder letter

First, this study shows that 34.80 % of the respondents have asked their doctors to check their prescriptions (Table 2). The average score of patient activation was 2.837, and the variable was dichotomized into high and low groups according to the mean score.

**Table 2:** Simple association between participants’ characteristics and response to the reminder.

Variables	Asked doctors to check patients' prescription				
	Yes	No	_X_^2^	*p value*	
**Patients asked doctors**					
Yes(n = 293)	293(34.84 %)				
No(n = 548)		548(65.16 %)			
**Patient activation**			34.347	<0.001	
Low**(n=634**)	186(29.33 %)	448(70.67 %)			
High**(n=207)**	107(51.69 %)	100(48.31 %)			
**Demographic/Socioeconomic**					
Age			5.982	0.05	
< 59 (n = 222)	36(31.86 %)	77(68.14 %)			
60–69 (n = 265)	83(30.07 %)	193(69.93 %)			
> 70(n = 354)	174(38.58 %)	277(61.42 %)			
Gender			0.04	0.841	
Male(n = 552)	191(34.60 %)	361(65.40 %)			
Female (n = 289)	102(35.29 %)	187(64.71 %)			
Income			3.099	0.212	
<NT$10000 (n = 70)	27(38.57 %)	43(61.43 %)			
NT$10000-NT$49999 (n = 428)	137(32.01 %)	291(67.99 %)			
>NT$50000 (n = 343)	129(37.61 %)	214(62.39 %)			
Education			2.963	0.277	
Primary school (n = 218)	69(31.65 %)	149(68.35 %)			
Junior high/Senior high (n = 355)	119(33.81 %)	233(66.19 %)			
College(n = 268)	105(38.75 %)	166(61.25 %)			
Marriage			2.192	0.139	
Married(n = 620)	207(33.39 %)	413(66.61 %)			
Single/Others (n = 221)	86(38.91 %)	135(61.09 %)			
**Health status**					
Reporting good or very good health			0.5624	0.453	
Yes (n = 474)	160(33.76 %)	314(66.24 %)			
No (n = 367)	133(36.24 %)	234(63.76 %)			
Hospitalized in the previous year			0.578	0.447	
No	77(37.02 %)	131(62.98 %)			
Yes	216(34.12 %)	417(65.88 %)			
A physician visit in the past month			0.001	0.974	
No	58(34.73 %)	109(65.27 %)			
Yes	235(34.87 %)	439(65.13 %)			
Number of medications you take every day			0.271	0.873	
1-2	60(33.90 %)	117(66.10 %)			
3-4	110(35.95 %)	196(64.05 %)			
5 or more	113(32.47 %)	235(67.53 %)			

Note: α = 0.05, ∗p < 0.05, ∗∗p < 0.01, ∗∗∗p < 0.001.

Among the participants with a high level of activation, 51.69 % had asked their doctors to check their prescriptions at their next clinic visit, which was higher than the rate of 29.33 % in the low activation group (p < 0.001). Other socio-demographic factors were not significantly associated with the reaction after receiving the reminder letter.

### 3.3 The effects of associated factors on patient activation and response to the reminder letter

Table 3 shows the results from the logistic regression models. The central column revealed the associated factors for a high level of patient activation. The results indicated that subjects aged 60–69 were less likely to have high activation scores (Odds ratio [OR] = 0.377, with a confidence interval [CI] 0.204–0.594) compared with those aged below 60 years. Subjects with high income greater than 50,000 NTD were more likely to have high activation scores (ORs = 1.637, CI 1.190–2.252. Subjects with higher education (college) were more likely to have high activation scores (OR = 1.619), yet subjects with a middle education level were less likely to have high activation scores (OR = 0.619). The results showed that health status measures were not associated with the level of activation.

**Table 3:** The effects of associated factors on patient activation and response to the reminder from logistic regression models.

Variables	High level of patient activation		Asked doctors to check patients' prescription	
	OR(95 % CI)	P value	OR(95 % CI)	P value
**Patient activation**				
Low			Reference	
High			2.617(1.880–3.642)	<0.001
**Demographic/Socioeconomic**				
Age				
< 59 (n = 222)	Reference		Reference	
60–69 (n = 265)	0.377(0.204–0.594)	<0.001	1.453(0.906–2.328)	0.121
> 70(n = 354)	1.099(0.778–1.551)	0.593	1.550(1.098–2.188)	0.013
Gender				
Male(n = 552)	Reference		Reference	
Female (n = 289)	1.086(0.773–1.528)	0.634	1.076(0.761–1.521)	0.679
Income				
<NT$10000 (n = 70)	Reference		Reference	
NT$10000-NT$49999(n = 428)	1.583(0.896–2.795)	0.114	0.936(0.535–1.638)	0.818
>NT$50000 (n = 343)	1.637(1.190–2.252)	0.002	1.238(0.896–1.710)	0.196
Education				
Primary school (n = 218)	Reference		Reference	
Junior high/Senior high (n = 355)	0.619(0.399–0.961)	0.033	1.662(1.047–2.639)	0.031
College(n = 268)	1.619(1.128–2.323)	0.009	1.163(0.813–1.663)	0.408
Marriage				
Single/Others (n = 221)	Reference		Reference	
Married(n = 620)	0.916(0.652–1.289)	0.616	0.697(0.494–0.983)	0.039
**Health status**				
Reporting good or very good health				
No (n = 474)	Reference		Reference	
Yes (n = 367)	0.965(0.702–1.325)	0.824	0.854(0.621–1.174)	0.331
Hospitalized in the previous year				
No	Reference		Reference	
Yes	0.967(0.679–1.376)	0.850	0.837(0.587–1.192)	0.324
A physician visit in the past month				
No	Reference		Reference	
Yes	0.881(0.603–1.286)	0.511	1.057(0.725–1.540)	0.774
Number of medications you take every day				
1–2	Reference		Reference	
3-4	1.122(0.746–1.686)	0.581	1.078(0.715–1.627)	0.719
5 or more	0.918(0.657–1.283)	0.617	0.993(0.708–1.392)	0.967

Note: α = 0.05, ∗p < 0.05, ∗∗p < 0.01, ∗∗∗p < 0.001.

Results in the right column show that respondents with a high level of patient activation were more likely to ask their doctors to check their prescriptions during the following visit (OR = 2.617, p < 0.001) than those with a low level of patient activation. The results also indicated that subjects over 70 years old, with a middle education level, and those with other marital statuses were more likely to respond to the reminder letter. Health status measures were not associated with the response to the reminder letter.

## 4 Discussion

The present study evaluated a nudge intervention of a postal reminder letter sent by an insurer to actively remind patients about medication duplication, thus attempting to motivate them to participate in the decision-making process for their medications. This study found that the postal reminder letter induced 34.84 % of the respondents to ask their doctors to check their prescriptions. From these results, a slight nudge in the form of a postal reminder letter can potentially encourage patients to take initiative in preventing prescription duplication.

This study also investigated associated factors affecting patients’ reaction to the reminder nudge, i.e., to ask the doctors to check their prescriptions. Alegría et al. [18] proposed that patient activation is one of the strategies to promote patient and doctor communication with each other. Existing research confirms that good communication between patients and physicians may be an important factor in patients taking a more active role in healthcare [30]. Our results align with prior literature, showing that respondents with high activation levels were more likely to take action (i.e., ask their doctors, 51.69 %) than those with low activation levels (29.33 %), as shown in Table 2. The regression models showed that patients with high activation level had higher odds (OR = 2.617) of asking the doctors to check their prescriptions at the next physician visit (Table 3). These findings suggest that higher patient activation might result in better health outcomes via communicating with doctors. Therefore, enhancing patient activation is one of the essential policies for improving the safety of prescriptions.

Relevant studies have identified that demographic and socioeconomic status, including age, education, and income, affect people's health behavior [[33], [34], [35]]. People with high educational levels also have higher health literacy than those with low educational levels [36]. The World Health Organization has proposed enhancing health literacy to maintain medication safety [37]. The public's health literacy can be cultivated by improving their health knowledge. Therefore, providing information regarding patients' medication safety to high-risk individuals via reminder letters is recommended.

The postal reminder letter intervention used in the present study was relatively low-cost (US$3 per person), which aimed to remind patients with medication duplication problems to ask their doctors to check their prescriptions. Traditionally, doctors made healthcare decisions by themselves; however, scholars have asserted that patients should participate in healthcare decision-making processes [38,39]. The present study revealed that postal reminder letters can induce patients to actively participate in healthcare processes and change the doctors’ prescribing behaviors, thus reducing inappropriate medication risks. Of course, prescription safety may be improved by computerized alert systems in healthcare settings [40,41]; yet the intervention was costly. Postal reminder letters may be considered as a supplementary measure for improving prescription safety and promoting patient participation.

The present study revealed that 65.16 % of patients did not ask the doctors to check their prescriptions after they received such postal reminder letters. We recommend that health authorities conduct multiple interventions to increase people's attention to and responsibility for personal health. Suppose other countries adopt postal reminder letters to improve doctors' healthcare behavior. In that case, the enhancement of patient activation must be incorporated to improve patients' health education, health literacy, and emphasis on personal health, thus improving the effectiveness of the intervention.

### 4.1 Limitations

This study has several limitations. First, the questionnaire survey yielded a relatively low response rate (7.84 %, 841/10,723), which may affect the representativeness of the sample and the generalizability of the findings. Nevertheless, this trial reminder letter project was the first attempt by the health insurance administration to nudge individuals and encourage active responses; thus, these preliminary results may still provide valuable insights for similar initiatives in other countries. Second, the low response rate may have introduced potential bias, as patients who completed the questionnaire might have exhibited a more proactive attitude toward their care, potentially influencing the study outcomes. Finally, patient activation was assessed using only three questions. Although prior studies have employed selected items to measure patient activation [42], including additional items could provide more reliable and valid measurements.

### 4.2 Conclusions

Health policy interventions on people's behavior changes may adopt financial incentives and technology-based nudge interventions. This study examined the effect of insurers' provision of medication safety reminder to the enrollees to drive healthcare providers to review and revise prescriptions. The results showed that using postal reminder letters as a nudging intervention tool can result in positive responses at a low cost. This study also found that patient activation is an important factor influencing patients' responses to the reminder. The findings from this study may be of value to health authorities and health insurers in other countries.

## Author contributions

YT: Formal analysis, Conceptualization, Software, Writing-original draft, Writing-review & editing. S-H C: Data curation, Investigation, Project administration, Review and editing.

## Data sharing

De-identified datasets analyzed in the current study are available from the corresponding author on reasonable request.

## Ethical statement

This study has been approved by the Institutional Research Ethics Committee of the National Taiwan University Hospital (201905067RINB).

## Funding

This study was partially supported by grants from the 10.13039/501100004737National Health Research Institutes in Taiwan (NHRI-EX112-11001PI). The funder had no role in the design, collection, analysis, and interpretation of data, in the writing of the manuscript, and in the decision to submit this manuscript for publication.

## Declaration of competing interest

The authors declare that they have no known competing financial interests or personal relationships that could have appeared to influence the work reported in this paper.
